# Growth, physiological responses, and meat quality of feedlot-finished Bonsmara steers offered unprocessed *Mucuna pruriens utilis* seed meal with or without conventional and green zinc oxide nanoparticles

**DOI:** 10.1007/s11250-024-04226-1

**Published:** 2024-11-12

**Authors:** Nozipho Phila Gamedze, Doctor Mziwenkosi Nhlanhla Mthiyane, Khomotso Sherdina Kgaswane, Sydney Mavengahama, Damian Chinedu Onwudiwe

**Affiliations:** 1 Department of Animal Science, School of Agricultural Sciences, Faculty of Natural and Agricultural Sciences, North-West University (Mahikeng Campus), Private Bag X 2046, Mmabatho, South Africa; 2 Food Security and Safety Focus Area, Faculty of Natural and Agricultural Sciences, North-West University, Mmabatho, 2735 South Africa; 3 North West Department of Agriculture and Rural Development, Private Bag X 804, Potchefstroom, 2530 South Africa; 4 Department of Crop Sciences, School of Agricultural Sciences, Faculty of Natural and Agricultural Sciences, North-West University (Mahikeng Campus), Private Bag X 2046, Mmabatho, South Africa; 5 Department of Chemistry, School of Physical and Chemical Sciences, Faculty of Natural and Agricultural Sciences, North-West University (Mahikeng Campus), Private Bag X 2046, Mmabatho, South Africa; 6 Material Science Innovation and Modelling (MaSIM) Research Focus Area, Faculty of Natural and Agricultural Sciences, North-West University (Mahikeng Campus), Private Bag X 2046, Mmabatho, South Africa

**Keywords:** Bonsmara, Mucuna seed meal, Conventional nanoparticles, Green nanoparticles

## Abstract

Feedlot finishing of beef cattle on commercial nutrient-dense diets based on expensive corn (maize), soybean meal (SBM) and other commonly used protein-rich ingredients is economically unsustainable, especially for smallholder farmers. Rich in energy and proteins, *Mucuna pruriens utilis* seed meal (MSM) could replace corn and protein-rich ingredients in beef cattle diets provided its problems of antinutritional factors (ANFs) and high fiber content that compromise animal growth performance are resolved. The objective of this study was, therefore, to investigate the effects of incorporation of conventional (C-Nano-ZnO) versus green (G-Nano-ZnO) zinc oxide (ZnO) nanoparticles in the diets of feedlot-finished Bonsmara steers supplemented with 20% MSM (dry matter basis). In a completely randomized design, 28 Bonsmara steers were randomly allocated to 4 experimental diets [i.e., Control diet without MSM (C); C with 20% MSM replacing corn (partially) and the common protein-rich ingredients (CM); CM with 25 mg/kg C-Nano-ZnO (CM-C); and CM with 25 mg/kg G-Nano-ZnO (CM-G)] each with seven replicates for 98 days. Performance variables, carcass traits, hemato-biochemistry, and meat quality were then measured. All data were analyzed with SAS using one-way ANOVA applying the GLM procedure, with diet as the independent variable, except for growth performance data that were analyzed as repeated measures. Results showed that while dietary MSM did not affect (*P* > 0.05) meat quality and serum biochemistry, it decreased body weight gain (BWG; *P* < 0.01), feed intake (FI; *P* = 0.001), feed conversion efficiency (FCE; *P* < 0.01), carcass fatness (*P* = 0.05), hot carcass weight (HCW; *P* < 0.05), cold carcass weight (CCW; *P* = 0.05), blood MCV (*P* < 0.05), MCH (*P* < 0.01), and neutrophils (*P* < 0.01) as it increased blood lymphocytes (*P* < 0.001). Interestingly, the harmful effects of MSM were attenuated by C-Nano-ZnO and worsened by G-Nano-ZnO. In conclusion, feeding of high dietary unprocessed MSM did not affect Bonsmara beef meat quality and serum biochemistry but compromised their growth performance, carcass traits, and some hematology responses, and these were alleviated by C-Nano-ZnO and deteriorated by G-Nano-ZnO incorporation. We recommend feeding commercial diets supplemented with 20% MSM, replacing corn and commonly used protein-rich ingredients, plus 25 mg/kg of C-Nano-ZnO to feedlot-finishing beef cattle.

## Introduction

Beef production plays a crucial role in the supply of highly nutritious food especially in developing countries with rapidly growing and malnourished human populations, including southern Africa. Indeed, a 100 g beef serving provides > 25% of the recommended dietary intake (RDI) of protein, selenium, zinc, niacin and vitamins B6 and B12, as well as > 10% RDI of iron, phosphorus, and riboflavin (Mwangi et al. 2019). Its protein supplies all the essential amino acids (Troy et al. 2016) plus antioxidants such as anserine and carnosine (Williams 2007; Asp et al. 2012). Also, beef fat supplies energy and fatty acids that contribute to meat flavor (Wyness et al. 2011). Hence, beef production holds the potential to help address the dire scourge of food insecurity and malnutrition in southern Africa where ~ 33% of the human population (~ 345 million) is severely food-insecure and 10% undernourished (FAO et al. 2019).

However, beef production in southern Africa is hampered by climate change and concomitant high feed costs. Climate change jeopardizes the availability and nutritional quality of forages in natural rangelands where most beef cattle are reared (Tavirimirwa et al. 2019; Vetter et al. 2020; Lamega et al. 2021). This situation has led to beef cattle farmers in the region adopting the global practice of feedlot finishing of pasture-backgrounded animals on commercial energy and protein-rich diets based on corn, soybean meal (SBM), cotton seed cake, sunflower seed meal, and other expensive, if not scarce, feed ingredients (DAF 2019; Beyzi 2020; Dezah et al. 2021; Sedibe et al. 2023). Consequently, there is a necessity to investigate more cost-effective alternative protein sources (APS) such as mucuna seed meal (MSM) for beef cattle diets.

*Mucuna pruriens utilis* (velvet bean: *Fabaceae* family) is a vigorously climbing annual legume indigenous to southern Africa (Trytsman et al. 2011). Its seeds (MSM) boast high levels of energy [(428–649 g crude carbohydrate per kg dry matter (DM)] and protein (230–430 g crude protein (CP) per kg DM) with a high content of essential and non-essential amino acids, minerals, and essential fatty acids (Ezeagu et al. 2003; Pugalenthi et al. 2005; Sowdhanya et al. 2024). The MSM also possesses hypoglycemic, lipid and cholesterol-lowering (Jayaweera et al. 2007; Dharmarajan and Arumugam 2012; Ngatchic et al. 2013; Ulu et al. 2018) as well as anabolic properties that enhance muscle mass and hence beef production (Lieu et al. 2012; Suresh and Prakash 2012; Yantika et al. 2016). *M. pruriens utilis* is highly adapted to elevated environmental temperatures and other abiotic stresses (Buckles et al. 1998), making it an ideal feed supplement for beef cattle in warm climates.

However, widespread usage of MSM as livestock feed is limited by its wide array of secondary metabolites, including alkaloids, amylase inhibitors, flavonoids, hemagglutinins, hydrogen cyanide, oxalates, phytic acid, saponins, trypsin inhibitors, tannins, and 3,4-dihydroxy-L-phenylalanine (L-DOPA) (Sowdhanya et al. 2024). While concentrations of most of these secondary metabolites do not cause detrimental effects in ruminant livestock (Szabo and Tebbett 2002), the major impediment to the extensive utilization of MSM as animal feed is its high concentration (≤ 11%) of L-DOPA (Pulikkalpura et al. 2015), an otherwise anti-venomous compound (Fung et al. 2011) that is used as treatment for Parkinson’s disease (Boniface et al. 2024). In this regard, dietary consumption of L-DOPA-containing MSM decreases dry matter intake (DMI) and body weight gain (BWG) in goats (Madzimure et al. 2014) and lambs (Chikagwa-Malunga et al. 2009a), despite rumen microbiota’s ability to biodegrade this toxic bioactive compound (Chikagwa-Malunga et al. 2009b). Another major constraint with MSM as animal feed is its high fiber content (97–193 g/kg DM), surpassing that of SBM (75 g/kg DM) and other legume seeds (Belewu and Olajide 2010). A high dietary fiber content prolongs chewing time and decreases ruminal degradation rate, feed passage rate, total nutrient digestibility, voluntary feed intake (FI) (Tafaj et al. 2005), and BWG (Jiyana et al. 2022) in ruminants. This circumstance accentuates the need for an innovative strategy to collectively address the problems of high fiber content, L-DOPA and other antinutritional factors (ANFs) in MSM prior to its use as beef cattle feed.

A growing number of studies have shown the potential of nanotechnology in enhancing the nutritional value of feedstuffs with high levels of fiber and ANFs. Indeed, nanoparticles can adsorb and improve the biodegradation of toxic chemicals (Asghar et al. 2020; Remya et al. 2022) as well as ruminal fiber degradation (Chen et al. 2011; Shi et al. 2011; Hosseini-Vardanjani et al. 2020). Synthesized from natural materials, mainly plant extracts, green (biogenic) nanoparticles are considered biologically safe and environmentally friendlier compared to conventional ones synthesized using toxic synthetic chemicals (Gamedze et al. 2024). Hence, it was hypothesized that dietary incorporation of green synthesized zinc oxide (G-Nano-ZnO) as compared to conventionally synthesized (C-Nano-ZnO) nanoparticles would enhance growth performance, carcass traits, hemato-biochemistry, and meat quality of beef cattle fed diets supplemented with a high level (20%) of MSM. Therefore, the objective of this study was to investigate the efficacy of G-Nano-ZnO versus C-Nano-ZnO in enhancing the nutritional utility of MSM as a replacement for corn grain and protein-rich ingredients (cotton seed cake, SBM, and sunflower seed meal) in the diets of feedlot-finished Bonsmara steers.

## Materials and methods

### Study site, source, and preparation of materials

The feeding trial was conducted using experimental pens at the North West Department of Agriculture and Rural Development in Potchefstroom (Topkraal) located at 26.73^0^ S, 27.07^0^ E. The synthesis, characterization, and validation of G-Nano-ZnO was performed in the Nanomaterials Laboratory of the Department of Chemistry, North-West University (NWU), whereas the C-Nano-ZnO was purchased from Merck Life Science (Pty) Ltd (South Africa). Mucuna seeds were obtained from AGT Foods Africa (Krugersdorp, South Africa) while all the feed ingredients were sourced from Simplegrow Agric Services Pty Ltd (Pretoria, South Africa).

### Chemical analysis

Milled samples of experimental diets and MSM samples were analyzed for proximate composition following the AOAC (2019) methods for DM (930.15), CP (984.13), crude fiber (978.10), ether extract (920.39) and ash (942.05). The neutral detergent fiber (NDF), acid detergent fiber (ADF), and acid detergent lignin (ADL) contents were determined using the ANKOM200/220 fiber analyzer (ANKOM Technology, Fairport, NY, USA) following the methods described by Van Soest et al. (1991). Minerals analyses were performed at the Centre for Applied Radiation Science and Technology at NWU. The samples were digested with 10 mL of 55% nitric acid for 48 min in an automated sample digester [Titan Microwave Sample Preparation System, PerkinElmer (Pty) Ltd]. Mineral concentrations were then determined using an inductively coupled plasma-mass spectrometer (ICP-MS) (Perkin-Elmer, 1982, NexION 300Q).

### Experimental design, diets, animal management, growth performance and slaughter

Ethical clearance and approval were acquired from the NWU (NWU-00804-22-A5) and the North West Department of Agriculture and Rural Development (NWAE/23/05/03). Based on their body weights, 28 Bonsmara steers (7–8 months old; 225.5 ± 17.80 kg) were randomly allocated to 4 experimental diets [i.e., Control Diet with 5% cotton seed cake, 4% SBM, and 1% sunflower seed meal (C); C with 20% MSM replacing yellow corn grain (partially) and the common protein-rich ingredients (CM); CM with 25 mg/kg C-Nano-ZnO (CM-C); and CM with 25 mg/kg G-Nano-ZnO (CM-G)], each with seven replicates for 98 days in a completely randomized design. Diets were formulated (Table 1) to meet the nutrient requirements of feedlot beef cattle according to NRC (2016) requirements. They were mixed fortnightly and safely stored in 50 kg bags in a feed shed at room temperature (25 °C).

**Table 1:** Ingredient and chemical composition (%) of experimental diets (DM basis) fed to Bonsmara steers

Ingredient	Dietary treatment				MSM
	C	CM	CM-C	CM-G	
Yellow corn grain	46.50	33.70	33.70	33.70	-
Rice bran	17.20	21.10	21.10	21.0	-
*Eragrostis* hay	12.30	11.00	11.00	11.00	-
Cotton seed cake, 40% CP	5.00	-	-	-	-
Soybean meal (SBM), 48% CP	4.00	-	-	-	-
Sunflower seed meal, 32% CP	1.00	-	-	-	-
*M. pruriens utilis* seed meal (MSM)	-	20.00	20.00	20.00	-
Lucerne meal	2.00	2.00	2.00	2.00	-
Molasses	8.00	8.00	8.00	8.00	-
Feed lime fine	2.00	2.00	2.00	2.00	-
Mono dicalcium phosphate	0.10	-	-	-	-
Salt fine	0.15	0.20	0.20	0.20	-
Urea	1.07	1.07	1.07	1.07	-
Acid Buf	0.38	0.38	0.38	0.38	-
Magnesium sulphate	0.23	0.45	0.45	0.45	-
Magnesium oxide	0.10	0.10	0.10	0.10	-
C-Nano-ZnO	-	-	0.025	-	-
G-Nano-ZnO	-	-	-	0.025	-
Monensin 20%	0.002	0.002	0.002	0.002	-
Calf grower premix	0.10	0.10	0.10	0.10	-
Total	100.00	100.00	100.00	100.00	-
*Chemical composition*					
Dry matter	87.240	88.595	88.595	88.595	90.005
Crude protein	13.918	13.900	13.900	13.900	23.269
Ether extract	4.761	7.466	7.466	7.466	3.049
Ash	4.350	4.044	4.044	4.044	2.487
Neutral detergent fiber	14.791	13.206	13.206	13.206	19.065
Acid detergent fiber	7.571	6.932	6.932	6.932	10.136
Acid detergent lignin	0.221	0.101	0.101	0.101	3.661
Calcium	1.200	1.200	1.200	1.200	0.007
Phosphorus	0.5	0.484	0.484	0.484	0.003
Potassium	1.248	1.315	1.315	1.315	0.066
Magnesium	0.289	0.285	0.285	0.285	0.001
Sodium	0.085	0.102	0.102	0.102	-
Chloride	0.350	0.350	0.350	0.350	-
Sulfur	0.192	0.250	0.250	0.250	0.004

C = Control diet; CM = Control diet with 20% MSM; CM-C = Control diet with 20% MSM and C-Nano-ZnO; CM-G = Control diet with 20% MSM and G-Nano-ZnO; C-Nano-ZnO = Conventionally synthesized zinc oxide nanoparticles; G-Nano-ZnO = Green synthesized zinc oxide nanoparticles

Before the commencement of the experiment, steers were vaccinated against clostridia (COVEXIN^®^ 10, Schering-Plough Animal Health Ltd., New Zealand, Reg. No: G3354), anthrax, botulism, and Black Quarter (Supervax^®^, Prondil S.A., Uruguay, Reg. No: G2643), dipped using Delete^®^ All (1mL/ 10 kg) for external parasites, and injected with Dectomax for internal parasites at the rate of 1 mL/50 kg body weight. They were also individually ear-tagged for identification and record-keeping purposes and weighed at the beginning of the trial and fortnightly after that to get BWG. The cumulative BWG data were based on initial and final BW (both with a 4% shrink application to account for digestive tract fill). Then the animals (without any implants) were placed in individual pens (2 × 2.5 m) each with a feeding and drinking trough and allowed to adapt to experimental diets for 14 days (total mixed ration *ad libitum*). At 08:00 h daily, feed and fresh clean water were offered to the animals, and the amounts of feed offered and leftovers in the feeding troughs were recorded for the duration of the experiment to calculate each animal’s FI. Feed conversion efficiency (FCE) was then calculated. The following formulae were used for calculating these variables:

a $$\:\text{B}\text{W}\text{G}\:(kg/d)=\frac{Final\:weight-Initial\:weight\:\left(kg\right)}{No.\:\:of\:days\:\left(d\right)}$$

b $$\:\text{F}\text{I}\:(kg/d)=\frac{Feed\:offered\:-\:feed\:left\:\left(kg\right)}{No.\:\:of\:days\:\left(d\right)}$$

c $$\:\text{F}\text{C}\text{E}=\frac{Body\:weight\:gain(kg/d)}{Feed\:intake\:(kg/d)}$$

On day 97, the steers were fasted overnight with access to drinking water. They were weighed on the morning of day 98, condition scored, and transported for slaughter at Syferfontein abattoir, about 10 km from the study site at Topkraal. Steers were humanely slaughtered in compliance with local regulations of animal welfare. They were electrically stunned with a dual point electrode placed on the head, bled (~ 2 min), skinned, and eviscerated. The head (sectioned at the atlano-occipital joint) and feet (sectioned at the metacarpal and metatarsal joints) were removed.

### Carcass measurements

Hot carcass weight (HCW) was determined after the removal of internal organs and perinephric–pelvic fat. The carcasses were kept at room temperature for 3 h for blood drainage before they were moved into a refrigerator (– 4 ^0^C). Cold carcass weight (CCW) and carcass length were measured at 24 h post-slaughter. The dressing-out percentage (%) was determined as a proportion of HCW to slaughter weight (Mapiye et al. 2009). Chilling loss was determined as reported by Simela et al. (2011) using the formula:

$$\:Chilling\:loss\:\%=\frac{HCW-CCW}{HCW}\times\:100$$

Carcasses were classified according to the official South African Carcass Classification System for age (by dentition) and fatness (visual appraisal) (DAFF 1990) and carcass conformation recorded. Carcass fatness was classified on a seven-class fat cover notation system (0, no visual fat cover; 1, very lean; 2, lean; 3, medium; 4, fat; 5, over-fat; and 6, excessively over-fat) by cutting through the skin in the loin area and measuring the subcutaneous fat with a ruler. Conformation was assessed on a scale of 1–5 (1, very flat; 2, flat; 3, medium; 4, round; and 5, very round). Carcass length was measured using a tape and expressed in meters.

### Meat quality analysis

Meat quality was post-mortem measured on the *Longissmus dorsi* muscle. The pH and temperature at 45 min and 24 h were measured using a Corning Model 4 pH-temperature meter (Corning Glass Works, Medfield, MA, USA) equipped with an Ingold spear-type electrode (Ingold Messtechnik AG, Udorf, Switzerland). Meat colour (L^*^ = lightness, a^*^ = redness, and b^*^ = yellowness) was measured using a Minolta colour-guide (BYK-Gardener GmbH, Geretsried, Germany), with a 20 mm diameter measurement area and illuminant D65-day light, 10° observation angle. Hue angle was calculated as tan^− 1^ (b/a), and chroma values were calculated using the equation$\:\:\sqrt{{a*}^{2}+{b*}^{2}}$. The muscles (40–50 g) were subsequently placed in a watertight plastic bag and cooked for 45 min in a water bath at 85 °C. Then, the samples were left to cool at room temperature and weighed again to determine cooking loss (AOAC 2016) using the following formula:

$$\eqalign{& {\rm{Cooking}}\>{\rm{loss}}\>\left( \% \right) = \cr & \,\,\,{{weight\>before\>cooking\left( g \right) - weight\>after\>cooking\left( g \right)} \over {weight\>before\>cooking\left( g \right)}} \times \>100 \cr} $$

After cooking, meat samples (5 × 5 cm) were cored parallel to the meat’s grain. Then they were sheared perpendicular to the fiber direction using a Warner Bratzler Meullenet-Owner’s razor shear blade mounted on a Texture Analyzer (Stable Micro System, Model: TA-XT plus, Surrey GU7 1YL, UK), and the cross-head speed was at 400 mm/min, with one shear in the centre of each core. The mean maximum load (N) was recorded.

Water holding capacity (WHC) was determined by placing muscle samples (5 ± 0.05 g) in-between two filter papers and placing a 60 kg weight on top for 5 min, followed by its calculation using the equation:

$$\:WHC\:\left(\%\right)=\:\frac{initial\:weight-weight\:after\:}{initial\:weight\:}\text{x}\:100$$

Lastly, drip-loss was determined by hanging the meat samples in air-tight glass bottle containers for 72 h, followed by its calculation using the equation:

$$\eqalign{& Drip\>loss\left( \% \right) = \cr & \,\,\,{{initial\>weight\>of\>sample - final\>weight\>of\>sample} \over {initial\>weight\>of\>sample}} \times \>100 \cr} $$

### Blood sampling and analysis

Blood samples for hemato-biochemical analysis were collected on the morning of the last day of the trial. Blood was collected from the tail vein by inserting an 18-guage vacutainer needle at about 30° angle. Five (5) mL of blood were collected from each steer into two sterilized tubes. The purple-top tubes containing EDTA as an anti-coagulant were used for hematology. In contrast, the red-top Vacuette^®^ Serum Clot Activator tubes without anticoagulant (Greiner Bio-One, GmbH, Frickenhausen, Germany) were used for serum biochemistry analysis. The purple-top tubes were inverted three times to mix the blood with the anticoagulant thoroughly. For hematological analyses, the samples were stored in a cooler box with ice, whereas for serum biochemical analyses, they were centrifuged at 3500 *g* for 10 min at 4 °C. The collected serum was stored at − 20 °C until further analysis. The hematological variables [hemoglobin, red blood cells, hematocrit, mean corpuscular hemoglobin (MCH), mean corpuscular hemoglobin concentration (MCHC), mean corpuscular volume (MCV), red blood cell distribution width (RDW), white blood cells, neutrophils, lymphocytes, monocytes, eosinophils, basophils, and mean platelet volume (MPV)] were determined using an automated IDEXX LaserCyte Hematology Analyser (IDEXX Laboratories, Inc. Johannesburg, South Africa). On the other hand, serum biochemical variables (calcium, phosphate, total bilirubin, alkaline phosphatase, alanine aminotransferase, amylase, lipase, total protein, albumin, globulin, cholesterol and glucose) were analyzed using an automated IDEXX Vet Test Chemistry Analyser (IDEXX Laboratories Inc., Johannesburg, South Africa).

### Statistical analysis

Data were analyzed using one-way ANOVA applying the general linear model (GLM) procedure of SAS (2012), with diet as the independent variable. They were then presented as Least Square Means (LSMs) with respective pooled standard errors of the means (SEMs) (*n* = 28). Where there were significant differences among treatments, the LSMs were compared by employing the probability of difference (PDIFF) option in the LSMEANS statement of SAS. The statistical model employed was: Y*ij* = *µ* + D*i* + Ɛ*ij*, where Y*ij* is the response variable, *µ* the population mean, D*i* the effect of diet, and Ɛ*ij* the residual error. Data with repeated measurements (FI, BWG and FCE) were analyzed using the GLM procedure of ANOVA with the employment of the statistical model: Y*ijk* = *µ* + D*i* + W*j* + (D×W)*ij* + Ɛ*ijk*, where Y*ijk* is the response variable, *µ* the population mean, D*i* the effect of diet, W*j* the effect of fortnight, (D×W)*ij* the effect of diet $\:\times\:$ fortnight interaction, and Ɛ*ijk* the residual error. For all the data, significance was declared at *P* ≤ 0.05 and a tendency between *P >* 0.05 and *P <* 0.1.

## Results

The effects of dietary MSM without or with C-Nano-ZnO and G-Nano-ZnO on overall and fortnightly performance variables of beef steers are presented in Table 2. While the steers had similar initial body weights (*P* > 0.05), dietary supplementation with MSM decreased their overall BWG (*P* < 0.01), FI (*P* = 0.001), and FCE (*P* < 0.01), with a tendency towards decreased body condition scores (*P* = 0.055), all of which were restored by C-Nano-ZnO and deteriorated by G-Nano-ZnO incorporation into experimental diets. Also, while there were no diet $\:\times\:$ fortnight interaction effects on FI (*P* > 0.05) and BWG (*P* > 0.05), there was a significant diet $\:\times\:$ fortnight interaction effect on FCE (*P* < 0.05), with dietary MSM inducing no effect on, and C-Nano-ZnO decreasing while G-Nano-ZnO increased, this economic variable only in fortnight 7.

**Table 2:** Fortnightly and overall growth performance and condition scores of Bonsmara steers fed diets supplemented without or with *M. Pruriens Utilis* seed meal and conventional (C-Nano-ZnO) and green (G-Nano-ZnO) zinc oxide nanoparticles (*n* = 7)

Variable	Fortnight	Dietary treatment				SEM	P-value		
		C	CM	CM-C	CM-G		Diet (D)	Fortnight (F)	**D** $\:\times\:$ **F**
Feed intake (kg/day)	1	7.360	6.731	7.083	5.622	0.631	0.001	0.01	0.563
	2	8.336	6.238	7.913	4.985				
	3	8.984	6.201	8.859	4.829				
	4	9.501	6.080	9.229	5.059				
	5	9.344	6.067	9.135	5.514				
	6	9.572	6.607	9.260	5.387				
	7	10.881	6.691	9.842	6.509				
Body weight gain (kg/day)	1	1.500	0.990	1.173	0.286	0.290	0.01	0.001	0.398
	2	2.071	0.663	1.571	0.798				
	3	1.878	0.776	1.684	0.333				
	4	0.898	0.561	1.010	0.204				
	5	1.381	0.969	1.418	0.469				
	6	0.762	0.510	0.816	0.5				
	7	4.857	3.020	3.633	3.238				
Feed conversion efficiency	1	0.204^a^	0.142^a^	0.159^a^	0.021^b^	0.039	0.01	0.001	0.031
	2	0.248^a^	0.094^c^	0.204^b^	0.148^b^				
	3	0.209^a^	0.122^b^	0.192^a^	0.051^b^				
	4	0.096^a^	0.092^a^	0.112^a^	0.009^b^				
	5	0.150^a^	0.157^a^	0.155^a^	0.045^b^				
	6	0.074	0.068	0.083	0.071				
	7	0.453^a^	0.448^a^	0.347^b^	0.507^a^				
Overall FI (kg)		9.05^a^	6.42^b^	8.77^a^	5.43^b^	0.65	0.001		
Overall BWG (kg)		1.67^a^	0.922^b^	1.48^a^	0.59^b^	0.18	0.002		
Overall FCE		0.18^a^	0.14^ab^	0.17^ab^	0.09^c^	0.02	0.003		
Body condition score		4.99	4.50	4.64	3.91	0.26	0.055		

^a,b,c^ Different superscripts within a row denote significant differences (*P* < 0.05). C = Control diet; CM = Control diet with 20% MSM; CM-C = Control diet with 20% MSM and C-Nano-ZnO; CM-G = Control diet with 20% MSM and G-Nano-ZnO; SEM = standard error of the mean

The effects of dietary MSM without or with C-Nano-ZnO and G-Nano-ZnO on carcass characteristics and meat quality of beef steers are presented in Tables 3 and 4. While there were no effects of diet on carcass conformation (*P* > 0.05), dressing % (*P* > 0.05), and chilling loss (*P* > 0.05), dietary MSM decreased carcass fatness (*P* = 0.05), HCW (*P* < 0.05), and CCW (*P* = 0.05), with a tendency towards decreased carcass length (*P* = 0.073), all of which were ameliorated by C-Nano-ZnO and deteriorated by G-Nano-ZnO inclusion in the diets (Table 3). Otherwise, diet had no effect on all meat quality variables (*P* > 0.05) (Table 4).

**Table 3:** Carcass characteristics of Bonsmara steers fed diets supplemented without or with *M. pruriens utilis* seed meal and conventional (C-Nano-ZnO) and green (G-Nano-ZnO) zinc oxide nanoparticles (*n* = 7)

Variable	Dietary treatment				SEM	*P-value*
	C	CM	CM-C	CM-G		
Carcass conformation	3.02	2.86	2.86	2.69	0.16	0.497
Carcass fatness	1.95	1.14	1.43	0.62	0.32	0.050
Carcass length (m)	1.83	1.75	1.77	1.71	0.03	0.073
Dressing %	56.94	57.13	54.91	60.93	2.02	0.227
Hot carcass weight (HCW; kg)	210.00^a^	170.00^b^	191.23^ab^	160.43^b^	11.22	0.023
Cold carcass weight (CCW; kg)	205.49^a^	165.82^b^	187.23^ab^	156.44^b^	10.97	0.020
Chilling loss (%)	2.15	2.47	2.05	2.53	0.23	0.336

^a,b^ Different superscripts within a row denote significant differences (*P* < 0.05). C = Control diet; CM = Control diet with 20% MSM; CM-C = Control diet with 20% MSM and C-Nano-ZnO; CM-G = Control diet with 20% MSM and G-Nano-ZnO; SEM = standard error of the mean

**Table 4:** Meat quality of Bonsmara steers fed diets supplemented without or with *M. Pruriens Utilis* seed meal and conventional (C-Nano-ZnO) and green (G-Nano-ZnO) zinc oxide nanoparticles (*n* = 7)

Variable	Dietary treatment				SEM	*P-value*
	C	CM	CM-C	CM-G		
pH_45min_	6.52	6.66	6.63	6.74	0.15	0.758
Temperature_45min_ (℃)	30.99	30.36	29.76	31.04	0.79	0.568
L^*^_45min_	27.11	28.54	29.07	28.07	0.64	0.166
*a* ^*^_45min_	9.39	9.03	8.59	8.02	0.41	0.121
*b* ^*^_45min_	0.41	0.55	0.32	0.76	0.16	0.178
H^*^_45min_	0.05	0.06	0.04	0.12	0.03	0.186
C^*^_45min_	9.62	9.35	8.82	8.93	0.30	0.214
pH_24h_	5.91	6.13	5.90	6.17	0.18	0.548
Temperature_24h_ (℃)	6.74	6.34	6.60	6.26	0.17	0.165
L^*^_24 h_	31.74	31.22	29.86	30.90	1.19	0.570
*a* ^*^_24 h_	8.26	9.94	9.53	10.31	0.75	0.233
*b* ^*^_24 h_	0.86	2.04	1.33	1.60	0.43	0.284
H^*^_24 h_	0.10	0.19	0.13	0.15	0.04	0.373
C^*^_24 h_	9.21	15.77	11.99	13.20	2.41	0.279
Drip loss %	2.52	2.38	2.44	1.71	0.40	0.473
Cooking loss %	39.69	35.23	38.14	35.61	1.72	0.219
Water holding capacity %	16.28	15.47	13.82	14.10	2.77	0.876
Moisture	71.27	71.85	72.95	74.17	1.11	0.269
Shear force (N)	6.29	6.56	6.31	6.49	0.15	0.484
Crude protein	34.54	36.11	37.34	36.28	0.85	0.146
Ether extract	1.79	1.97	2.18	1.96	0.35	0.730
Ash	2.15	2.45	2.13	2.55	0.40	0.762

C = Control diet; CM = Control diet with 20% MSM; CM-C = Control diet with 20% MSM and C-Nano-ZnO; CM-G = Control diet with 20% MSM and G-Nano-ZnO; L* = lightness; a* = redness; b* = yellowness; C* = chroma; H* = hue angle; SEM = standard error of the mean

The effects of dietary supplementation with MSM without or with C-Nano-ZnO and G-Nano-ZnO on the hemato-biochemistry variables of steers are shown in Tables 5 and 6. Diet did not affect hematological (*P* > 0.05) and biochemical (*P* > 0.05) variables except for the MSM-induced decrease in MCV (*P* < 0.05), MCH (*P* < 0.01), and neutrophils (*P* < 0.01) in contrast to increased serum lymphocytes (*P* < 0.001). In a pattern reciprocating lymphocyte responses, all the decreases in the hematological variables were alleviated by C-Nano-ZnO and worsened by G-Nano-ZnO inclusion into the animal diets.

**Table 5:** Hematology of Bonsmara steers fed diets supplemented without or with *M. Pruriens Utilis* seed meal and conventional (C-Nano-ZnO) and green (G-Nano-ZnO) zinc oxide nanoparticles (*n* = 7)

Variable	Dietary treatment				SEM	*P-value*
	C	CM	CM-C	CM-G		
Hemoglobin (g/dL)	11.87	11.30	11.39	11.23	0.78	0.946
Red blood cells (10^12^/L)	8.05	8.65	7.70	8.31	0.66	0.692
Hematocrit (%)	37.43	36.24	35.66	36.23	2.72	0.986
MCV (fL)	46.59^a^	41.84^c^	46.60^ab^	43.99^abc^	1.33	0.034
MCH (pg)	14.79^a^	13.10^b^	14.89^a^	13.65^ab^	0.42	0.009
MCHC (g/dL)	31.74	31.31	31.99	31.06	0.44	0.454
RDW (%)	21.37	22.01	21.53	23.06	0.79	0.396
White blood cells (10^9^/L)	10.98	10.07	10.92	9.78	1.21	0.839
Neutrophils (%)	20.26^ab^	17.10^ab^	24.19^a^	12.94^b^	1.90	0.004
Lymphocytes (10^9^/L)	60.77^b^	68.53^a^	55.59^b^	70.30^a^	2.17	0.0003
Monocytes (10^9^/L)	15.01	12.01	15.86	13.69	1.29	0.127
Eosinophils (10^9/^L)	2.77	1.04	2.97	1.97	0.72	0.198
Basophils %	1.20	1.31	1.40	1.10	0.45	0.668
Mean platelet volume (fL)	8.52	7.84	7.66	8.35	0.29	0.103

^a,b,c^ Different superscripts within a row denote significant differences (*P* < 0.05); C = Control diet; CM = Control diet with 20% MSM; CM-C = Control diet with 20% MSM and C-Nano-ZnO; CM-G = Control diet with 20% MSM and G-Nano-ZnO; MCH = mean corpuscular hemoglobin; MCHC = mean corpuscular hemoglobin concentration; MCV = mean corpuscular volume; RDW = red blood cell distribution width; SEM = standard error of the mean

**Table 6:** Serum biochemistry of Bonsmara steers fed diets supplemented without or with *M. Pruriens Utilis* seed meal and conventional (C-Nano-ZnO) and green (G-Nano-ZnO) zinc oxide nanoparticles (*n* = 7)

Variable	Dietary treatment				SEM	*P-value*
	C	CM	CM-C	CM-G		
Calcium (mmol/L)	2.37	2.40	2.37	2.29	0.06	0.434
Phosphate (mmol/L)	3.38	3.15	3.28	3.22	0.22	0.829
Total bilirubin (umol/L)	5.28	5.27	4.43	5.44	0.77	0.720
Alkaline phosphatase (U/L)	160.83	161.29	199.57	158.43	29.60	0.570
ALT (U/L)	33.74	27.39	26.71	23.58	3.15	0.460
Amylase (U/L)	126.62	102.76	110.86	120.78	6.70	0.612
Lipase (U/L)	13.50	15.86	9.86	11.190	4.27	0.061
Total protein (g/L)	61.87	62.14	65.71	62.01	1.86	0.193
Albumin (g/L)	34.86	32.29	34.43	31.86	1.09	0.131
Globulin (g/L)	27.49	29.86	31.29	30.16	1.56	0.324
Cholesterol (mmol/L)	2.92	3.27	2.97	2.89	0.25	0.618
Glucose (mmol/L)	3.73	3.50	4.00	3.47	0.24	0.319

C = Control diet; CM = Control diet with 20% MSM; CM-C = Control diet with 20% MSM and C-Nano-ZnO; CM-G = Control diet with 20% MSM and G-Nano-ZnO; ALT = alanine aminotransferase; SEM = standard error of the mean

## Discussion

This study investigated the efficacy of C-Nano-ZnO versus G-Nano-ZnO in enhancing the utilization of MSM as replacement for yellow corn grain and protein-rich ingredients in feedlot-finishing diets of Bonsmara cattle. Including 20% MSM adversely affected beef steers’ growth performance and carcass characteristics. Our findings corroborate those of Madzimure et al. (2014); Chikagwa-Malunga et al. (2009a) who respectively found decreased DMI and BWG in indigenous goats, as well as BWG in lambs, fed MSM-supplemented diets. Also, the current results align with those of Yantika et al. (2016) where, despite an increase in DMI, BWG and FCE decreased in finisher bulls fed a diet with 16% MSM. Interestingly, these researchers observed no ill effects on cattle performance variables when their diet was supplemented with 12% of the APS. Evidently, a level of MSM in the diet ≥ 16% accumulates an excess of L-DOPA, which decreases FI, leading to reduced growth rate and FCE. It is postulated that the observed decreased FI was induced by L-DOPA conversion to dopamine, a precursor of epinephrine (adrenaline) (Meiser et al. 2013), a stress-related catecholamine that induces anorexia (Joshi et al. 2022; Wallace and Fordahl 2022). Epinephrine could have induced the anorexic effect either alone or in concert with other catecholamines dopamine and/or norepinephrine (Leibowitz 1978). Indeed, catecholamine-releasing drugs including L-DOPA can inhibit feeding (Hoebel 1985) and dopamine alone can independently inhibit FI by activating peripheral dopamine D2 receptors (Reinholz et al. 2008; Johnson and Kenny 2010). The decrease in FI induced by epinephrine or other catecholamines would have caused growth hormone deficiency (Nakagawa et al. 1985), leading to compromised growth performance and carcass characteristics of steers, similar to previous observations (Cuneo et al. 1992; Carroll et al. 1998).

The significant diet $\:\times\:$ fortnight interaction effect on FCE, with no adverse effect of MSM on this parameter only in fortnight 7 of the study, suggests time-dependent attainment of adaptation to consumption of L-DOPA by the steers. This response corroborates previous studies demonstrating time (age) dependent achievement of adaptation to the toxicity of various ANFs in ruminants (Dhillon et al. 1971; Sidhu et al. 1996; Soto-Blanco 2008). Mechanistically, the ruminal microbiota eventually adapts to biodegrade L-DOPA after prolonged exposure to this bioactive compound, as previously reported by Chikagwa-Malunga et al. (2009b). Similarly, prolonged feeding of a saponin-rich feed was shown to terminate the deleterious effects of the ANF on ruminal microbiota (Wina et al. 2006). The putative underlying mechanism involves ruminal microbiota attaining adaptation to antinutritional dietary phytochemicals by increasing the thickness of their cell walls (Wang et al. 2000).

On the other hand, this is the first study to demonstrate the detrimental effects of dietary MSM on HCW and CCW, with a tendency towards decreased carcass length, in beef cattle. Hence, there are no comparable studies in the literature. Nevertheless, these findings are unexpected as MSM is known to enhance meat (carcass) yield through L-DOPA’s anabolic and muscle accretion effects (Lieu et al. 2012) as well as the capacity of ruminal microbiota to biodegrade the toxic bioactive compound extensively (Chikagwa-Malunga et al. 2009a, b). This response implies that with MSM at 20% (DM basis) in the diet, the L-DOPA concentration would have overwhelmed the ruminal microbiota’s ability to biodegrade this phytochemical. Consequently, L-DOPA, instead of enhancing muscle accretion, apparently induced toxicity to the steers. This response would imply that the 14-day adaptation period of steers was probably too short to get the ruminal microbiota of the cattle adapted to biodegrade L-DOPA.

However, the observed decreased carcass fatness and lack of detrimental effects on other carcass characteristics, particularly chilling loss, and meat quality in response to dietary MSM are of great interest. In this regard, modern consumers generally prefer lean beef, considering the predominance of saturated fats in bovine meat (Harfoot and Hazlewood 1988), which are associated with chronic diseases (Judd et al. 2002). Also, the lack of effect of dietary MSM on chilling loss is highly desirable as it suggests the potential utility of MSM in retaining the meat’s moisture. Indeed, we observed a lack of effect of dietary MSM on the WHC of beef. This response is a highly desirable attribute of MSM, considering the capacity of WHC to directly influence other meat quality variables (Jennen et al. 2007; Jung et al. 2016; Wen et al. 2020) as well as its low values being associated with the pale, soft, exudative (PSE) meat and high values with dark, firm, dry (DFD) meat (Gonzalez-Rivas et al. 2020). We believe it is due to the lack of effect of dietary MSM on important meat quality attributes such as pH and WHC that the legume supplement did not influence all the meat quality variables measured in this study. This response corroborates the findings of Yantika et al. (2016) who also observed no effects of feeding diets supplemented with 12% and 16% MSM on pH, cooking loss, WHC, and tenderness in meat.

Hemato-biochemistry indices are essential indicators of the pathophysiology of the animal and a clinical tool for monitoring their health, especially when fed a diet containing toxic ANFs (Raimi and Adeloye 2022). Hence, the observed lack of effect of diet on most hematological and serum biochemical variables suggests that dietary supplementation with MSM did not compromise the health and physiology of steers, with all reported values within normal ranges for finishing beef steers (Carrillo et al. 2021). Notwithstanding, MSM decreased MCV, MCH, and neutrophils, suggesting that the APS induced iron deficiency anemia in the steers. In this regard, MCV and MCH (in addition to MCHC) are red blood cell indices that respectively determine the size of the cells and define their amount of hemoglobin (Sarma 1990; Åsberg et al. 2014). In a previous study, iron deficiency anemia (IDA) co-occurred with low hemoglobin, MCV, ferritin, and neutrophils (neutropenia) (Abdelmahmuod et al. 2020), and such IDA-associated neutropenia was reversible with iron therapy (Yassin et al. 2022). Indeed, diets with unprocessed MSM in rats increased hemoglobin distribution width (HDW), with a tendency towards increased MCHC and cellular hemoglobin concentration mean (CHCM), indicative of IDA (Huisden et al. 2019). While our data indicate MSM-induced IDA in the steers, blood iron concentrations were not measured in this study. Otherwise, the mechanism underlying the increased serum lymphocytes in response to dietary MSM in the steers remained elusive. However, this could have been induced by MSM’s L-DOPA, in line with Hurny et al. (2013), who observed appreciably increased CD16 and CD19 lymphocytes in the peripheral blood of Parkinson disease patients treated with L-DOPA.

The observed beneficial effects of C-Nano-ZnO in terms of animal performance, carcass characteristics, and hematology suggest that the conventional nanomaterials can bind noxious phytochemicals in MSM or enhance their biodegradation, as well as that of dietary fiber, by the ruminal microbiota. Due to their high surface area, nanoparticles possess an enormous ability to bind toxic chemicals with greater adsorptive capacity compared to conventional binding agents (Puzyr et al. 2007; Gibson et al. 2011). This attribute of nanoparticles would render MSM’s L-DOPA and other phytochemicals ineffective in causing toxicity to the steers. In addition to the nanomaterials’ immune-stimulating and antimicrobial properties, this would also account for their previously observed ability to improve growth performance and health in animals (Reda et al. 2020; Michalak et al. 2022). Also, nanoparticles possess the ability to enhance ruminal fiber degradation (Chen et al. 2011; Shi et al. 2011; Mohamed et al. 2015, 2017; Hosseini-Vardanjani et al. 2020), which would enhance the digestibility and utilization of dietary nutrients as well as ruminal production of volatile fatty acids (Adegbeye et al. 2019), leading to improved animal growth performance, carcass yield, and hematology as depicted in C-Nano-ZnO-induced increases in BWG, FI, FCE, body condition scores, carcass fatness, carcass length, HCW, CCW, MCV, MCH, and neutrophils. However, the mechanism underpinning the observed C-Nano-ZnO-induced decrement, in contrast to the G-Nano-ZnO-induced rise, in blood lymphocytes could not be explained.

Meanwhile, it would appear from the observed deleterious effects of G-Nano-ZnO on animal performance, carcass traits, and hematology that, contrary to expectations, the biogenic nanoparticles unleashed significant toxicity to the steers. These green nanoparticles were synthesized using the extract of mucuna seeds as a source of phytochemicals for the reduction and stabilization of Nano-ZnO (Gamedze et al. 2024). With the most toxic phytochemical in MSM being L-DOPA (Huisden et al. 2019), it is speculated that the biogenic nanomaterials were probably encapsulated with the noxious bioactive compound, which would account for their detrimental effects on BWG, FI, FCE, body condition scores, carcass fatness, carcass length, HCW, CCW, MCV, MCH, and neutrophils of steers. Otherwise, G-Nano-ZnO may have induced toxicity to the cattle through different mechanisms, as described before (Dmochowska et al. 2020; Machado et al. 2021; Zavitri et al. 2023). In this regard, the nanoparticles have been reported to induce toxicity to the intracellular environment through their dissolution into free Zn^2^^+^ ions and formation of reactive oxygen species, leading to oxidative stress (von Hellfeld et al. 2020) and inflammation (Pandey and Mishra 2022). Unfortunately, this study did not measure biomarkers of oxidative stress and inflammation to provide a mechanistic explanation of the harmful effect of G-Nano-ZnO.

However, the observed G-Nano-ZnO-induced increase in contrast to C-Nano-ZnO-induced decrease in steer FCE in fortnight 7 suggests that the bovine animals attained adaptation to metabolizing the toxic green nanomaterials upon prolonged exposure to them. This circumstance may have occurred similarly to the observed time-dependent adaptation to L-DOPA toxicity, as discussed above. Seemingly, G-Nano-ZnO also behaved like an ANF, with the animal’s ruminal microbial biodegrading systems getting accustomed to the nanomaterial upon prolonged exposure to it, as it was previously observed with dietary secondary metabolites (Wina et al. 2006; Soto-Blanco 2008). Indeed, a previous study demonstrated that silver nanoparticles affected short-term memory but not long-term memory in rats (Hritcu et al. 2011), probably through prolonged bovine exposure to G-Nano-ZnO decreasing the concentration of L-DOPA-derived dopamine, as observed with manganese nanoparticles (Hussain 2006). However, the observed C-Nano-ZnO-induced decrease in steer FCE in fortnight 7 could not be explained and there are no comparable studies in the literature that report dietary effects of nanomaterials on FCE responses of steers fed MSM-supplemented diets.

## Conclusion

Dietary inclusion of 20% unprocessed MSM (DM basis) in finishing cattle diets as replacement for corn grain and commonly used protein sources compromised growth performance and carcass characteristics without affecting hemato-biochemistry and meat quality of Bonsmara steers. Interestingly, dietary incorporation of C-Nano-ZnO generally ameliorated, while G-Nano-ZnO worsened the harmful effects of MSM.

## Data Availability

Data will be made available upon reasonable request.
